# Systems approaches in osteoarthritis: Identifying routes to novel diagnostic and therapeutic strategies

**DOI:** 10.1002/jor.23563

**Published:** 2017-04-24

**Authors:** Alan J. Mueller, Mandy J. Peffers, Carole J. Proctor, Peter D. Clegg

**Affiliations:** ^1^ Faculty of Health and Life Sciences Department of Musculoskeletal Biology Institute of Ageing and Chronic Disease University of Liverpool William Henry Duncan Building, 6 West Derby Street Liverpool L7 8TX United Kingdom; ^2^ The MRC‐Arthritis Research UK Centre for Integrated Research into Musculoskeletal Ageing (CIMA) Liverpool United Kingdom; ^3^ Institute of Cellular Medicine Newcastle University Framlington Place Newcastle upon Tyne NE2 4HH United Kingdom

**Keywords:** osteoarthritis, systems biology, cartilage, modelling

## Abstract

Systems orientated research offers the possibility of identifying novel therapeutic targets and relevant diagnostic markers for complex diseases such as osteoarthritis. This review demonstrates that the osteoarthritis research community has been slow to incorporate systems orientated approaches into research studies, although a number of key studies reveal novel insights into the regulatory mechanisms that contribute both to joint tissue homeostasis and its dysfunction. The review introduces both top‐down and bottom‐up approaches employed in the study of osteoarthritis. A holistic and multiscale approach, where clinical measurements may predict dysregulation and progression of joint degeneration, should be a key objective in future research. The review concludes with suggestions for further research and emerging trends not least of which is the coupled development of diagnostic tests and therapeutics as part of a concerted effort by the osteoarthritis research community to meet clinical needs. © 2017 The Authors. *Journal of Orthopaedic Research* Published by Wiley Periodicals, Inc. on behalf of Orthopaedic Research Society. J Orthop Res 35:1573–1588, 2017.

Osteoarthritis (OA) has been recognized in the earliest forms of man, and throughout animal species, located wherever there is a diarthrodial articular surface. Yet, as we approach 275 years since William Hunter's description of ulcerated cartilage as “a very troublesome disease”^1^ the therapeutic strategies available range from benign neglect to whole joint replacement. OA cannot be considered a single disease with a linear narrative describing its pathogenesis, rather it is a heterogenous condition of multiple causation with a degenerate, non‐functional joint the common end‐point.^2^ Subject to repetitive cycles of loading over many years the joint represents the functional product of integrated multisystem, multiphysics, and multiscale units.^3^ The objective of this review is to consider afresh whether the osteoarthritis research community has tackled the need for novel OA therapeutics and diagnostics by applying recent developments in systems biology. Suggestions are made for areas of research that require further development and methods, which have shown utility in other disciplines, as described. We consider mechanotransduction in osteoarthritis as a systems orientated case‐study, but there are no OA studies that demonstrate the iterative and cyclical process of testing, validation, and refinement consistent with a systems biology approach. Additionally, we wish to consider why, given the decades of research and prevalence of OA,^4^ that there are still no disease modifying therapeutics or prognostic markers and how progress should proceed with respect to trends and regulatory frameworks. Not all tissues contributing to OA are well‐represented in systems orientated studies and where possible pertinent examples are provided.

## BIOLOGY AS A SYSTEM

A biological system is a set of elements (e.g., genes, proteins, and metabolites) with multiple and diverse functions; these elements interact in a specific and non‐linear manner to produce coherent behaviors over time. Evolution has defined specialized interactions creating functional systems and sub‐systems at the cell, tissue, organ, organismal, and population/ecological levels.^5^ Critically, the functional nature of the system is neither a characteristic of single elements, or only of the interactions of these elements; rather, behavior arises from a combination of these characteristics. The Human Genome Project demonstrated that biology is an information science. Information is hierarchical^6^ and this structure is replicated in biological systems (DNA‐mRNA‐proteins). Therefore, complexities inherent to biological systems must be addressed using computational solutions as traditional reductionist strategies, intuition, and cognitive capacity alone will not be sufficient to develop a predictive understanding of biological systems and their derangements.^7^ It is the primary purpose of a systems biology approach to harvest high‐quality data in a systematic and comprehensive manner from all levels of the biological hierarchy and integrate this data with the intention of developing predictive models of the system. With this objective in mind it is necessary to consider that not only is the quality of the data variable, but often incomplete and biased. Genes of unknown function, or unknown interacting partners, are often ignored and emphasis is often placed on those most studied. Functional annotations relating to musculoskeletal disease, especially OA, are poor and result in spurious descriptors. These important issues have been realized^8^ and methods to improve annotations are being developed.^9^

### Systems Biology: A Paradigm Shift in Science

**Figure 1:**
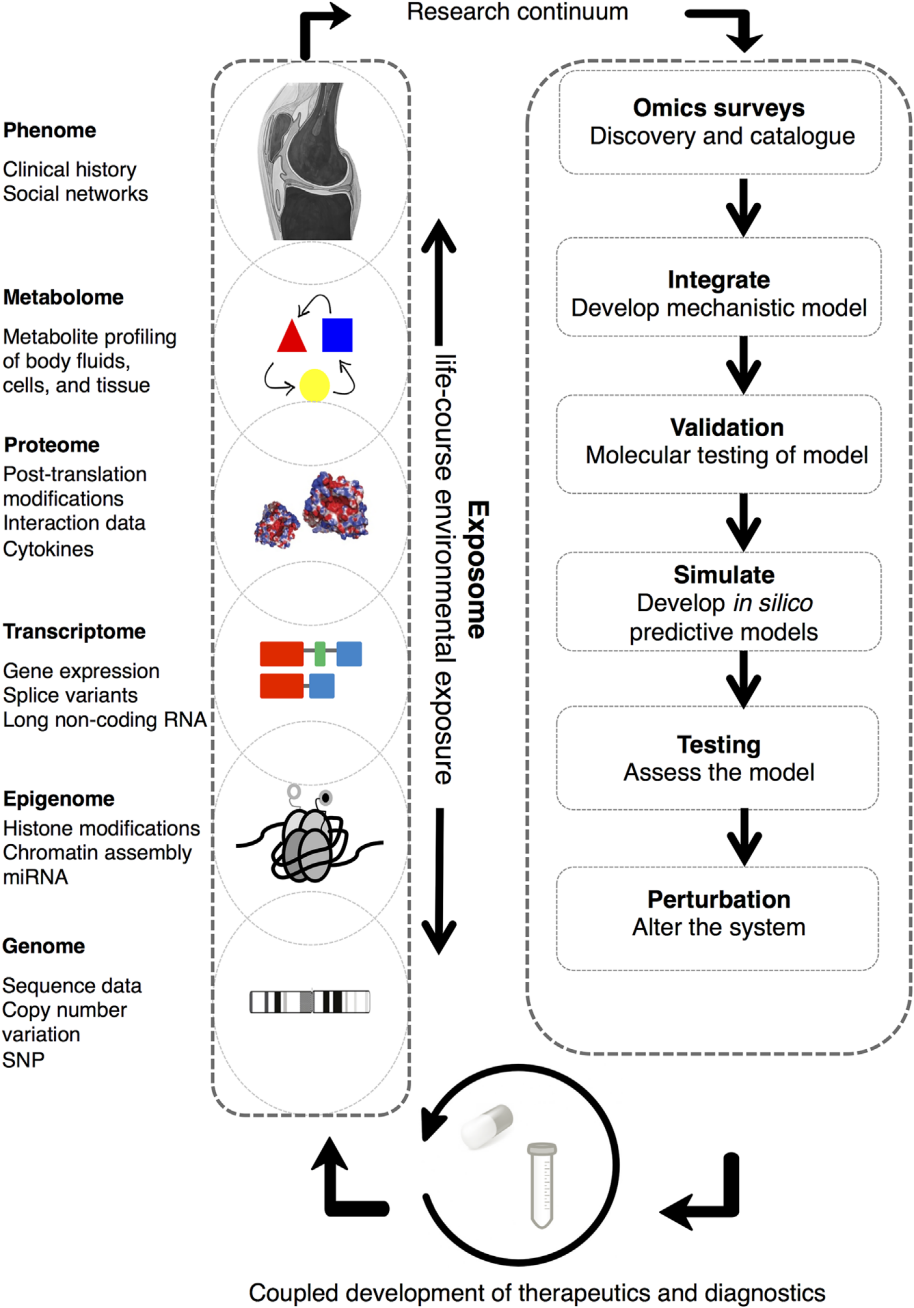
The iterative systems biology approach to defining novel diagnostic and therapeutic targets. Schematic demonstrates a prototypical, multi‐stage, systems orientated approach to develop novel diagnostic and therapeutic solutions to a complex disease problem such as osteoarthritis. Not all options may be applicable in every study. Omics surveys are depicted as intersecting “snap‐shots” of the biological hierarchy. Recursive profiling of the biological hierarchy is relevant in systems‐orientated approaches as it may reveal: (a) patterns of activity and isolated structures repeated at different levels; (b) information at one hierarchical level may not represent activity at another; (c) multi‐directional causality, that is, information passing both within and between levels in the hierarchy; and (d) non‐locality of function; functional activity may occur distant to other system elements (e.g., synapses in a neuron, actin filaments at the leading edge of a cell). The integration of these elements is critical to the development of mechanistic models; this may include defining scales by which to couple levels or use approaches that span scales (e.g., phenotype and gene expression). The exposome defines an individual's cumulative risk factors over their life (e.g., obesity, joint trauma). Validation at the molecular level may give insights into regulatory principles to produce initial in silico simulations. Testing the simulation, perturbing the system, and subsequent re‐profiling are further elements of the cycle. The co‐development of OA diagnostics and therapeutics is consideration within this process. Given the considerable time and resources that are required to sustain this continuum suggests that community‐orientated approaches using standardized methodologies are essential. Subsequently, patient feedback, adverse events, data from mobile health technologies, can be incorporated into iterative rounds of improvement. The review demonstrates that in the last decade studies have only considered elements of this continuum, for example ‘omics surveys. In general, studies are incomparable limiting that capacity to integrate. Figure developed from concepts introduced in refs.^113^, ^114^

Fundamental definitions and frameworks for a systems approach have been well‐described^6^, ^10^, ^11^, ^12^, ^13^ and are covered briefly with respect to OA research, Figure 1. In this review we consider “systems orientated”^7^ approaches to OA; frequently OA studies do not fulfill the requirements of a holistic systems biology approach. Systems orientated studies may begin without a clear hypothesis and are often agnostic to pre‐existing knowledge of molecular biology. This initial stage comprehensively catalogues the elements present in the system under investigation and consists of single or multi‐omics surveys (transcriptome, proteome, epigenome, metabolome). Time is an important component of this approach and dynamic observations should be made. Much of the contemporary OA literature has achieved the first stage, however, a systems biology approach must proceed with an iterative series of systematic perturbations and quantifications to measure elements from all the distinct levels of a biological system. In an attempt to recapitulate the behavior of the system all the quantitative data must be integrated into a network model. This mathematical model is reconciled with observed responses then a new hypothesis is formulated and tested experimentally. It is not the purpose of this review to assess the extent to which an OA study conforms to the ideals of a systems biology approach rather recognize the contribution each study makes toward such an approach, and define the gaps in our understanding of OA pathogenesis. In time this should aid the design of future studies with view to ultimately establishing OA diagnostic tests and therapeutics.

### Complexity in Osteoarthritis

**Figure 2:**
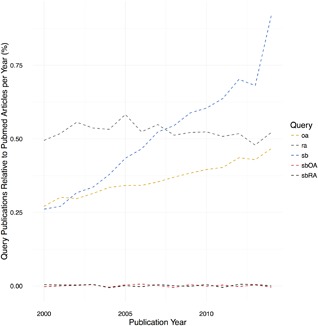
Publication trends associated with the following query terms: “rheumatoid arthritis” (ra), “osteoarthritis” (oa), “systems biology” (sb), or combinations of these terms (sbOA and sbRA) expressed as a percentage of the total number of publications (https://www.ncbi.nlm.nih.gov/pubmed) per year (2000–2014). Trend lines for sbOA and sbRA have been “jittered” to avoid over‐plotting. Data for 2015/16 are incomplete and are not included. Publications associated with OA have grown slowly with respect to RA; in contrast systems biology publications have shown a rapid increase in the decade following the publication of the Human Genome (2001) to represent ∼0.9% of publications in 2014. Publications referencing either OA or RA and systems biology still account for a very small contribution to the total number of annual publications (0.001%).

OA is the most prevalent chronic joint disease and the most common co‐morbidity of the ageing population. Incidence increases with age and is also associated with other predisposing factors such as obesity and joint trauma.^14^ The biomechanical failure of articular cartilage, together with changes in other joint tissues, demonstrates that OA is a whole joint disease as early changes are also evident in subchondral bone^15^ and synovium.^16^ OA should be considered a complex disease; the disease phenotype is a consequence of the interplay between heterogeneous and multiple genomic variants, dysfunctional regulatory systems, and environmental contributions with spatiotemporal distributions.^17^, ^18^, ^19^ The identification of genes responsible for common Mendelian traits, by linkage and linkage disequilibrium analysis,^17^ has not been possible for OA; defining causative mutations from phenotypic associations has demonstrated few candidate risk loci. While insidious degeneration results in a common end‐point, a non‐functional articulation, the initiating causes or mechanisms are often unclear. For the homeostasis or health of the joint stability of the system arises as a function of the integrated behavior of the biological, mechanical, and structural elements of the system.^20^ In Chu et al,^21^ an apt analogy is made between the probability of developing OA and the alignment of biological, mechanical, and structural factors as a slot/fruit machine. With each of these factors, and other associated risks, there is a probability of incitement of *pre*‐osteoarthritis as the homeostatic mechanism becomes dysregulated. The early consideration of the abnormal characteristics of these components, and inclusion of known prior risk factors promoting a propensity to OA, would be useful in determining at risk groups. When considered in this way it is clear that the historical focus on the end‐stage OA phenotype has distracted from recognising the relationship between all factors. Despite understanding that the inciting factors are likely to be heterogeneous we still recognize similar disease phenotypes; this suggests that at least some common elements of the system are likely to be dysregulated at some stage.^18^ Fundamentally, those elements that preserve the homeostatic system are still poorly understood. Using systems biology approaches it becomes possible to understand how these elements interact or infer the missing nodes. Through understanding how the homeostatic system responds to perturbations, rational approaches to diagnostic tests and therapeutic development can be made. When considering publications since the turn of the century, explicitly considering systems biology and OA, only a small increase in investigations in this field in recent years is evident, Figure 2.

### Systems Orientated Studies Exploring OA

In the interest of brevity notable systems orientated studies in OA will be considered generally as “top‐down” (‘omics integration, network‐based, metabolic, and image‐based studies) and “bottom‐up” (dynamical models and molecular and pathway analyses). The studies are chosen as examples of the research objectives associated with a systems orientated approach to OA.

### In Vitro Models—Routes to Regeneration and Cell Therapies

Tissue engineering and regenerative therapies are a major focus of attempts to modify the progression of OA.^22^ In general, much of the in vitro basic OA research, in particular for chondrocytes, is still undertaken using monolayer or well‐established three‐dimensional culture models. Two transcriptomic studies have considered the underlying mechanisms associated with differentiation transitions for in vitro chondrocytes using systems approaches. By understanding the regulatory mechanisms of de‐ and re‐differentiation transitions chondrocytes may be manipulated in tissue‐engineering and regenerative medicine. Work from our group defined mechanistic networks associated with phenotypic transitions in two‐ and three‐dimensional culture systems relative to native cartilage.^23^ Revealing gene expression in chondrocytes at the single cell level Cote et al.^24^ found considerable cell‐to‐cell heterogeneity in gene expression both in chondrocytes and mesenchymal stem cells under directed differentiation toward a chondrocytic phenotype. Both studies have implications on our understanding of how chondrocytes may be manipulated (directed differentiation) in cell‐based regenerative therapies for OA and the validity of current mechanistic models using established laboratory approaches. An obvious future approach would be the application of stochastic modeling techniques to quantify the biological variability and uncertainty in single cell measurements^25^, ^26^; failing to consider this may influence the interpretation of in vitro experiments.

### Network Medicine

Interaction networks may be generated from the elements of a biological system; abstractions of these networks can facilitate an understanding of the architecture, activity, and key players in that system. Much like a spider's web a perturbation in one part of a network will be propagated throughout. Network medicine postulates a “disease module” hypothesis, where disease‐associated genes or proteins are likely share the same topological neighborhood in a network. Defining communities of network elements (genes, proteins) is a useful way to identify elements that have a close relationship, shared functionality, or disease association. A systems biology approach to comprehending OA is founded on the hypothesis that OA is a multi‐system disorder resulting from the dysfunction of a number of networks that, together, alter the homeostatic balance of the joint. Therefore, comprehensive and multisystem approaches are necessary to understand the complexity of OA and direct the development of innovative treatment strategies. To date most studies pertaining to using a systems approach in OA research are principally based on interrogation of a single ‘omics survey in a single tissue at a single time point. A reference set for transcriptomic and proteomic studies is provided in Table 1. Genome‐wide association studies in OA are reviewed elsewhere.^27^, ^28^, ^29^

**Table 1:** Example Studies Using Systems Biology Approaches in OA Relevant Samples Applicable to Osteoarthritis Research

	Sample					
Question	Type	Origin	Species	Goal	Principle platform(s)	Reference
Descriptive	Tissue	Cartilage	Human	OA; intact and damaged transcriptome	RNASeq	Dunn et al.^35^
Descriptive	Tissue	Cartilage	Human	OA secretome	Mass spectrometry proteomics, relative quantification	Lourido et al.^115^
Descriptive	Tissue	Cartilage	Human	OA secretome	Mass spectrometry proteomics, absolute quantification	Peffers et al.^116^
Descriptive	Cells	Chondrocytes	Equine	Ageing transcriptome	RNASeq	Peffers et al.^117^
Descriptive	Cells	Chondrocytes	Human	OA post transcriptome	Microarray	Tewet al.^118^
Descriptive	Cells	Chondrocytes	Human	OA methylome	Methylation arrays	Rushton et al. 119
Descriptive	Cells	Chondrocytes	Human	OA genetic loci	GWAS	Evangelou et al. 120
Descriptive	Tissue	Cruciate ligament	Human	Sex‐related proteome	Mass spectrometry proteomics, relative quantification	Little et al.^121^
Descriptive	Fluid	Synovial fluid	Horse	OA	Mass spectrometry proteomics, relative quantification	Peffers et al.^122^
Descriptive	Fluid	Synovial fluid	Human	OA metabolome	NMR metabolomics	Zhang et al.^123^
Descriptive	Tissue	Subchondral bone	Rat	OA transcriptome	Microarray	Zhang et al.^124^
Descriptive	Organ	Joint	Mouse	Age and OA transcriptome	Microarray	Loeser et al.^125^
Integrative	Cells	Cartilage, tendon	Rat	Transcriptomic changes in culture	Microarray	Mueller et al.^23^]
Integrative	Cells	Bone‐marrow derived MSCs	Human	Transcriptome and methylome ageing	RNASeq, methylation array	Peffers et al.^126^
Integrative	Tissue	Synovial	Human	OA Transcriptome and proteome	Microarray	Lorenz et al.^127^
Integrative	Organ	Joint	Mouse	OA time course	Microarray	Olex et al. 33
Integrative	Cells	Chondrocytes	Human	OA	Microarray and protein microarray	Illiopoulos et al. 128
Pertubation/model testing	Cells	Chondrocytes	Human	OA microRNA	miRNASeq	Crowe e al.^129^
Computer model‐led	Organ	Joint	Mouse	Age	Computer modelling	Hui etal.^39^
Computer model‐led	Cells	Chondrocytes	Human	Cartilage breakdown	Computer modelling	Proctor etal.^130^
Computer model‐led	Cells	Chondrocytes	Human	Cytokine response	Computer modelling and proteomics	Melas etal.^36^
Computer model‐led	Cells	Periosteal derived stem cells	Human	Chondrocyte hypertrophy	Computer modelling and gene expression analysis	Kerkhofs et al.^41^
Computer model‐led	Organ	Knee joint	Human	Assessing surgical treatments for osteoarthritis	Computer modelling, MRI of knee joints	Mootanah etal.^131^
Computer model‐led	Organ	Brain (endocannabinoid system)	Human	Pain response in osteoarthritis	Computer modelling	Benson et al.^132^
Computer model‐led	Organ	Knee joint	Human	Stresses in response to cartilage overloading	Computer modelling	Mononen et al.^58^

### Network‐Based Systems Orientated Studies

Network‐based approaches make use of known or inferred functional and physical interactions between the elements of a system or can be developed from statistical associations (e.g., correlations between expression values) a priori giving a high‐level understanding of the organization of the system.^18^ Data are often collected from disparate sources and organized into a coherent structure that can be interrogated by graph theory or logical (probabilistic) approaches.^25^ Additionally, they can be applied in a flexible manner to multi‐omics and clinical data, and across scales. Several studies have used network‐based approaches to define communities of molecules that share the same neighborhood within a network as molecules implicated in OA. Work by Nacher et al.,^30^ made use of the Google PageRank algorithm to define novel disease candidate proteins that share a network neighborhood with known OA proteins. These high‐ranking proteins were derived from an interactome constructed from multiple proteomic studies of chondrocytes. The assumption is that membership by novel candidate proteins of an OA‐associated sub‐network means they are more likely to be subjected to the same perturbations. The small, ubiquitin‐related modifier SUMO4 was shown to interact with 15 OA‐associated proteins with the main interacting partners related to glycolysis and redox regulation. Using existing protein‐protein interaction data and an automated sub‐network searching tool (jActiveModules^31^) Loeser et al., defined a sub‐network associated with genes up‐regulated during the initial 4 weeks after destabilization of the medial meniscus (DMM) in a murine model, including heparin‐binding EGF‐like growth factor (HB‐EGF).^32^ Olex et al.^33^ developed this strategy further using time‐course gene expression data from a whole mouse joint model of OA to define perturbed sub‐networks. ECM‐receptor interaction and focal adhesion canonical pathways were enriched across all time‐points. This approach facilitated an understanding of the global phasic changes in expression of classic OA‐associated genes following joint trauma.

Protein–protein interactions represent compound data arising from many cell types and biological contexts so may not be indicative of the biological system under investigation and so generic networks without biological specificity may arise. Soul et al.^34^ developed an integrated tool (PhenomeExpress) to define context‐specific sub‐networks in an unbiased manner. This method utilized prior knowledge of cross‐species phenotype‐to‐gene connections to establish sub‐networks containing differentially expressed genes describing associations with a phenotypic correlation in the disease of interest. The largest sub‐network identified was annotated with immune function terms consistent with an understanding of pro‐inflammatory changes in sub‐chondral bone in OA. Further work by this group^35^ using the PhenomeExpress approach in a small, paired RNA‐seq analysis of OA cartilage versus normal sites defined several sub‐networks associated with *Wnt*‐signaling, apoptosis, matrix organization, and mitotic cell cycle.

Other network approaches have included the use of Boolean dynamics to consider the coupled sequential reactions (signal propagation) between elements of a pathway to define a mechanistic network that was predictive of the response of primary chondrocytes to different ligands, including those associated with OA pathophysiology.^36^ Our own work has included the use of weighted gene co‐expression analysis (WGCNA)^37^ to define sub‐networks of highly connected genes (modules) that have strong associations with sub‐groups of human osteoarthritic cartilage. We demonstrated cross‐species preservation of system development and immune‐associated modules between gene expression profiles from human OA and rodent models.^38^ As these examples perhaps confirm, the frequency with which comparable key regulators and functional descriptors arise in these studies may be attributable to the data bias previously described.

### Mechanistic Studies and Dynamic Models

The limitation of many network approaches is that they require mapping of expression data onto pre‐existing protein interactions and so rely heavily on prior knowledge of signalling and metabolic pathways. Furthermore, statistical associations are made with respect to end‐stage disease phenotypes, rather than having pre‐osteoarthritis as the focus of investigations. Critically, network approaches cannot capture spatiotemporal, dynamic changes in the system. Generally, network approaches in OA have been useful in identifying novel targets and sub‐networks, however, further mechanistic evaluation, perturbation, simulation, or validation of the proposed sub‐networks are performed infrequently. Complex disease phenotypes change with time and are subject to biochemical and biophysical fluxes. Often, the signals of interest may be spatially constrained, or example cell‐matrix interface. Network models cannot capture this and so require to be coupled to dynamic models to provide a description of how a system progresses both in space and time. Only a few studies have considered this for chondrocytes or with respect to OA. Using observed immunohistochemical changes in cartilage from ageing mice and candidate proteins associated with cartilage destruction and ageing Hui et al., developed an integrated computational model accounting for progressive collagen loss and increased MMP13 production.^39^ By modulating pathway elements the study demonstrated oxidative stress and the IL‐1 pathway were integral to progressive loss of cartilage matrix. Notably, the model predicted differential temporal expression of MMP13 through the simulated inhibition of IL‐1 or ALK1. This approach is more useful than descriptive ‘omics studies for developing a detailed, tissue‐specific, mechanistic understanding and simulating temporal responses to perturbations facilitating rational hypothesis development for further testing. Both network‐based and molecular approaches provide useful insights into the pathophysiology of OA, but have not been used as part of a systems‐biology continuum. Kerkhofs et al.^40^ developed a mathematical model to examine the switch from resting/proliferating chondrocytes to hypertrophy.^41^, ^42^ The systems approach included a form of Markov chain model to predict the probability of particular factors pushing a chondrocyte toward a proliferative (*Sox9*) or hypertrophic (*Runx2*) phenotype. There is currently a dearth of validated dynamic, mechanistic models and a critical need to link these “bottom up” studies to the network models generated by “top‐down” approaches.

### Constraint‐Based Models of Metabolism

An understanding of the metabolic derangements associated with the joint tissues contributing to OA, and their molecular context, would be invaluable to defining pathogenic pathways especially given the evidence of whole‐body metabolism effects on OA risk.^43^ A number of contemporary studies have provided useful reference metabolomic and proteomic data from osteophytic cartilage,^44^ subchondral bone,^45^ and synovial fluid,^46^ or considered the role of metabolic pathways derived from transcriptomics data.^33^, ^47^ However, our understanding of the homeostatic control of metabolic fluxes in cartilage, bone, and synovial fluid is limited. Constraint‐based (CB) models facilitate a large‐scale understanding of metabolic fluxes without the necessity for detailed kinetic information (e.g., from Hui et al.^39^), which is often lacking. Information on the stoichiometry of all the metabolic reactions is considered within a pseudo‐steady state that is optimized for a particular “objective function”; methods include *metabolic flux* and *flux‐balance analysis* (FBA). There are few examples in the literature relating to constraint‐based approaches to modelling metabolic fluxes in joint tissues and no large‐scale FBA simulations, including gene‐knockout simulations, have been carried out for OA associated tissues. In Salinas et al.,^48^ the authors used metabolic flux analysis to determine the changes in central metabolism pathways in chondrosarcoma‐derived SW1353 cells in response to mechanical loads. Although this study makes a novel contribution to our understanding only limited metabolic pathways are considered. Furthermore, it becomes difficult to attribute metabolic changes arising from transduced mechanical signals to pro‐matrix synthesis pathways. The limitations of the FBA approach relates to the inability to incorporate dynamic information or regulatory elements. This requires the integration of ‘omics data into generic genome‐scale metabolic reconstructions^49^, ^50^ to generate cell‐ and tissue‐specific models. Generic metabolic reconstructions serve as templates for more specific contexts. High quality genome‐scale generic human metabolic models are freely available and have recently been revised.^51^ Considerable resources (e.g., COBRA Toolbox 2.0,^52^) have been made available to generate context‐driven tissue/cell‐specific metabolic models. This has been successfully performed either in a draft or high‐quality model form for many cells and tissues^50^ but, musculoskeletal tissues are poorly represented (skeletal muscle,^53^ foetal cartilage^54^), if not absent from these analyses.

Unlike constraint‐based metabolic flux analyses of micro‐organisms a metabolic understanding of OA requires the construction and coupling of metabolic networks for multiple tissues from the same organism. Common interactions may be defined by metabolites that are secreted or consumed between tissues, but defining these elements, the post‐transcriptional modifications that govern tissue‐specific metabolic activity profiles,^53^ and the extent to which this coupling occurs in vivo is a considerable challenge. In the case of micro‐organisms, or neoplastic tissues, the functional objective is growth. In trying to develop a multi‐scale model of the articular joint in the adult human the functional activities of each tissue will be distinct from growth, but likely to have an optimization or efficiency objective.^49^ Practical frameworks for the development of these context‐driven models are available^50^; it should be a priority in osteoarthritis research to develop joint tissue‐/cell‐ specific metabolic models. Overall, there is a necessity to make use of the available data to define cell‐/tissue‐specific metabolic models that incorporate molecular information from ‘omics studies. Large‐scale simulation and perturbation studies using CB analysis should be undertaken. Gene knock‐outs can by simulated in tissue‐specific models to direct further molecular validation of regulatory mechanisms.^55^ Methods to infer missing or unidentified metabolites in untargeted metabolomic studies, incorporating network techniques, will facilitate a tissue‐specific understanding.^56^ These studies, in due course, will provide the input to the development of coupled, multi‐tissue whole joint metabolic models. Such projects are on the scale of those undertaken for the liver, brain, and kidney or for particular diseases (e.g., diabetes); as such, they will require collaborative efforts.

### Image‐Based Physiological Models

Physiological models derived from advanced imaging techniques (computed tomography (CT), micro‐CT, magnetic resonance imaging (MRI), and in vitro techniques, e.g., quantitative microscopy) may be used to simulate musculoskeletal systems and are useful approaches to developing a systems understanding of OA. The data are derived directly from the applicable study group, physiological conditions may be applied in a repeatable manner, and temporal changes may be simulated. Predictions of the material properties of the constituent tissues may be made that could not otherwise be easily measured experimentally; multiple tissues, or specific tissue elements, may be considered in their physiologically relevant setting. The approach is non‐destructive and tissue‐failure conditions may be estimated in a non‐invasive manner. The temporal impact of pathology or treatment can be simulated in the model. Overall, these approaches are cheaper, faster, and knowledge‐driven compared to in vivo models. The integration of high‐resolution geometry available from advanced imaging techniques and constraints defined by biomechanical data may be used to develop finite element simulations of the joint tissues. Although these imaging techniques have been more widely applied to muscle and bone this modelling approach is uncommon for cartilage and sub‐chondral bone in the context of OA although some reports are found in the literature. For example, using high‐resolution micro‐CT of the mouse tibia it has been possible to estimate the mechanical characteristics of the femoro‐tibial joint DMM surgery using finite element analysis (FEA).^57^ The dynamic structural damage that occurs at the articular cartilage, which would otherwise be difficult to test, was explored in silico. Mononen et al.^58^ also used finite element modelling to simulate cartilage degeneration using MRI data of knee joints from normal weight and obese OA patients. Using a functional imaging approach to reveal bone metabolism Hirata et al.^59^ correlated changes in ^18^F‐fluoride PET (positive‐emission tomography) uptake with stress distributions in the subchondral bone of coxo‐femoral joints from patients. These few examples suggest that there is still considerable work required to link clinical or functional measurements with in silico models for a number of OA‐associated tissues. There are efforts to develop standardized, open‐source finite element joint models,^60^ but this requires not only to capture the variation in human anatomy, mechanics, and kinematics, but they are also required for model species where the majority of basic studies will be validated.

### Multiscale Modelling

**Figure 3:**
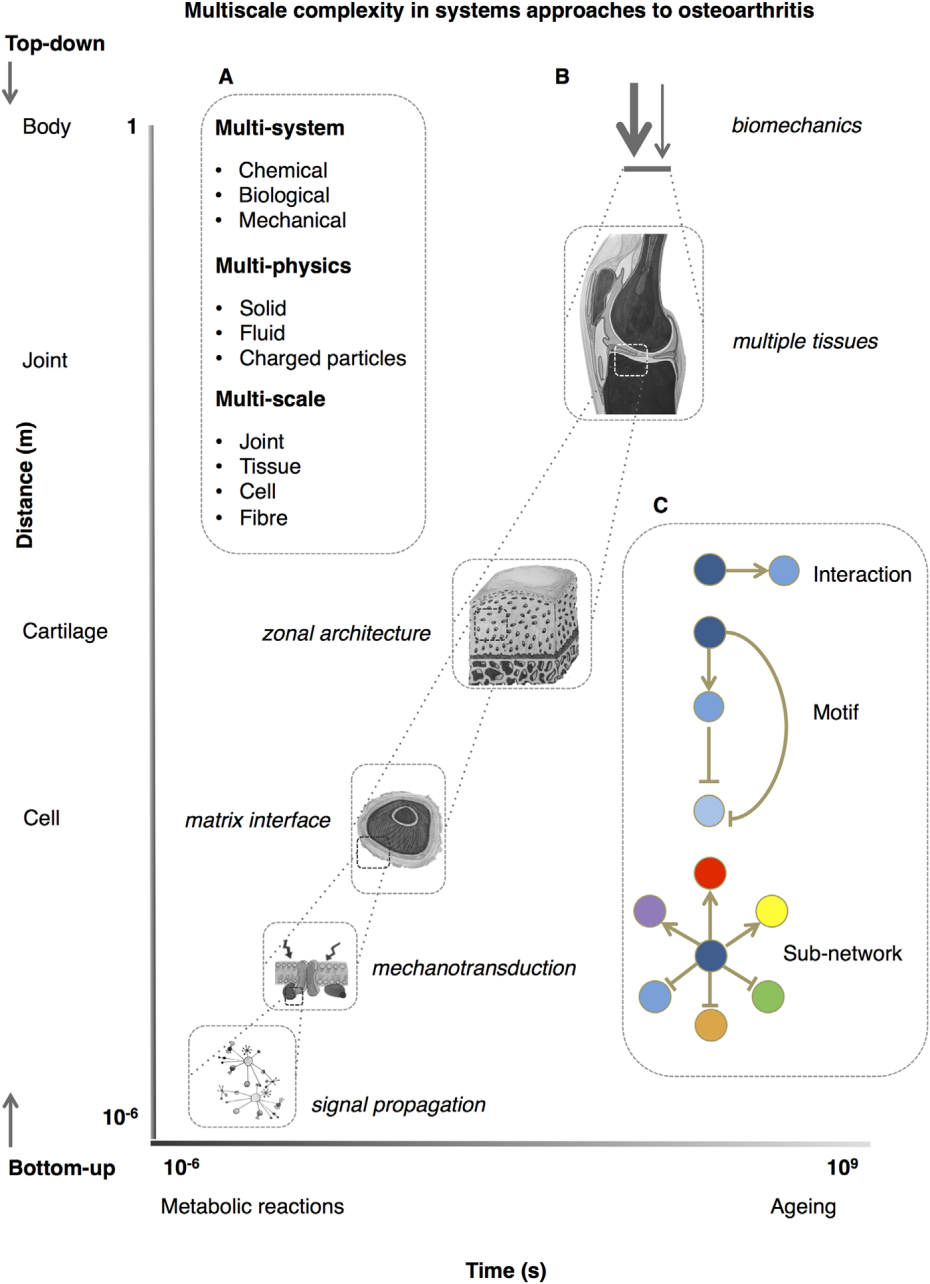
Multiscale complexity in developing systems models in osteoarthritis. (A) Selecting and defining the appropriate sub‐system for analysis is critical in a systems‐orientated approach to complex diseases such as osteoarthritis. Osteoarthritis presents multi‐scale, ‐system, and ‐physical ‐system, and‐physical problems. Approaches may be considered “top‐down” or “bottom‐up,” though in practice this is not a sequential process with many studies adopting a “middle‐out” approach. (B) Coupling scales and integrating data across levels of the biological hierarchical is non‐facile when attempting to derive useful prognostic, predictive, or therapeutic outputs. Osteoarthritis is presented as a series of spatiotemporal problems. For example, time ranges from microsecond interactions in metabolic reactions to the course of human longevity, collagen turnover, and the requirement for a functional joint. Spatially, anatomy, load sharing, propagation of mechanical signals, and localized responses at interfaces show considerable breadth. (C) Network scales range from gene networks to social networks. The component of a network that is being considered is important, whether this is a simple interaction, a regulatory motif, or multiple sub‐networks. Additional complexity arises from a diverse phenome and inciting factors, stochasticity in gene expression, and non‐local events. The use of animal models adds a layer of complexity to this problem and appropriate regard must be given to the spatial and temporal differences in these models. Figure developed from concepts described in refs.^3^, ^21^, ^61^, ^63^

**Figure 4:**
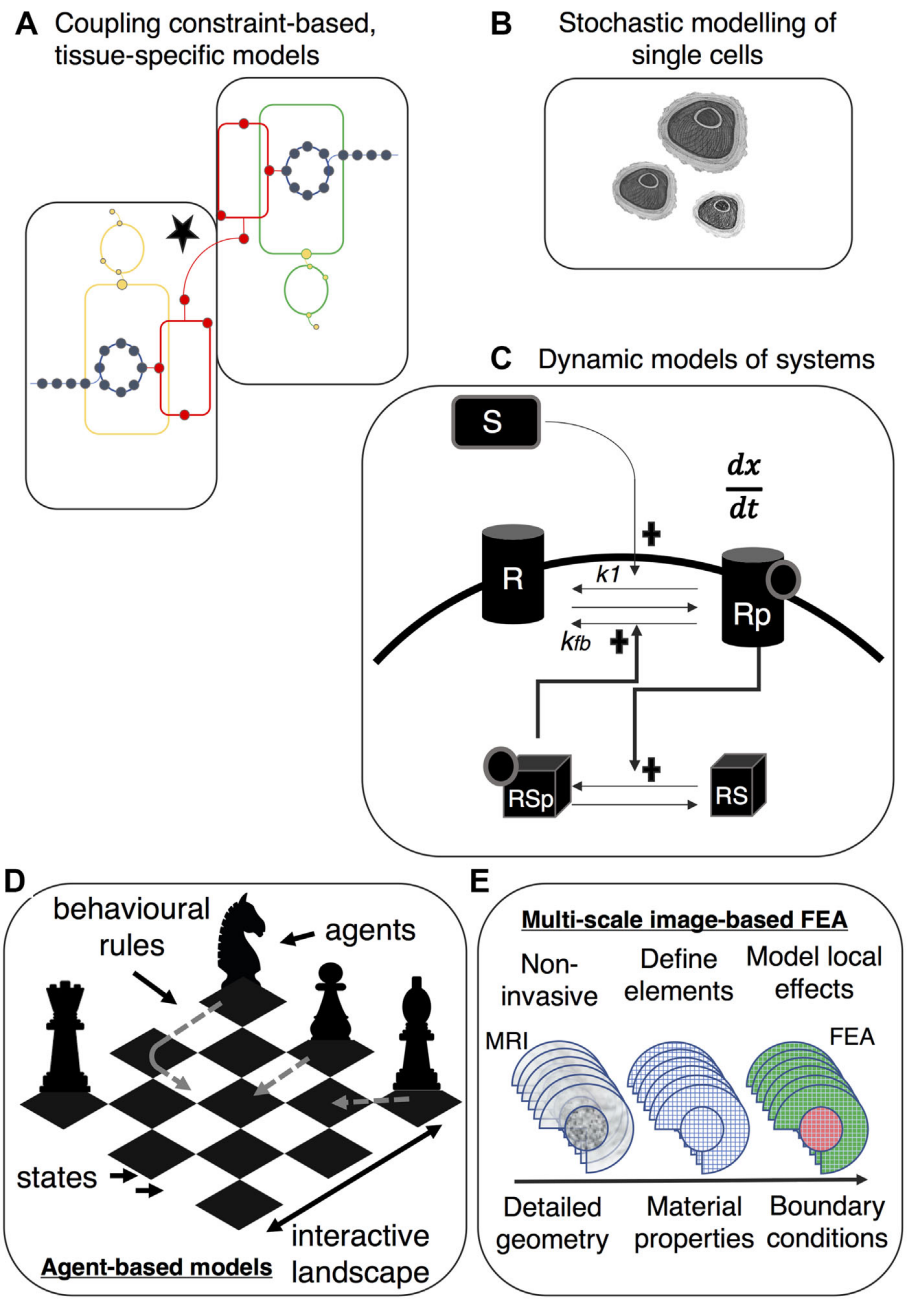
Future strategies for systems orientated studies in osteoarthritis—(A) Coupling constraint‐based, tissue‐specific metabolic models requires the identification of metabolites that are shared across systems,^49^ for example sub‐chondral bone and cartilage; simulation of single or multiple gene knock‐outs^55^ has also yet to be explored (B) Most analysis of cellular behavior occurs at the population level and considers average responses. More finely‐grained appreciation of cellular behavior, such as spatially restricted signaling, requires stochastic modelling at a single‐cell (or sub‐cellular) level.^26^ (C) Most network models are static and have not been validated. Dynamic models, using a series of ordinary differential equations, may be used to simulate a hypothetical regulatory mechanism (e.g., positive feedback), which may then inform in vitro validation studies; (D) Agent‐based modelling is a “bottom‐up” approach that uses the activity and interactions of autonomous “agents” (e.g., cells) to simulate and predict the observed complex behavior. In this schematic the interaction space and potential states are depicted by a chess board where agents are represented by tchess pieces; each has rational constraints on its behavior. Decision‐making heuristics and learning processes may be applied to simulate the complex behavior of the system. Such approaches have been frequently applied to multi‐scale problems^67^ and may be applicable to modelling the complex behavior within, and between, tissues.^25^ (E) Highly detailed geometric information derived from advanced‐imaging techniques and material properties can be used in finite‐element models of multiple musculoskeletal tissues.^62^

The purpose of multiscale models is to develop early patient‐orientated intervention packages based upon a realization of trauma risk, the predicted performance of an intervention, and the prognostic capacity of biomarkers or clinical measurements as proxies for cell‐level responses.^3^, ^61^ As we have highlighted in the sections above, there are approaches to integrating high‐throughput data into tissue‐coupled, constraint‐based metabolic models, and across scales for mechanical studies,^62^ but this is not yet a common approach in OA. Additionally, there is no evidence of clear ‘omics integration approaches relevant to OA in the literature. To make significant progress in our understanding of OA pathogenesis, metabolic, and biomechanical models will have to be coupled across multiple temporal and spatial scales, Figures 3 and 4. Biological systems already integrate all this information, however, for researchers this is a non‐trivial concern with a large number of complex modelling and data integration techniques available.^63^, ^64^ Ageing and sex manifests as anatomical and mechanical changes^65^ that must also be integrated into multiscale models. It is evident that there is still insufficient basic structural and molecular understanding of the elements of OA‐associated tissues to fully realize multiscale approaches at this time. One alternative strategy that offers a way to approach multiscale problems and simulate complex systems behavior is agent‐based modelling (Fig. 4).^25^ The activity and interaction of autonomous “agents” (e.g., cells), consisting of simple behavioral rules, may be formulated to simulate the collective behavior of these agents. As yet, this is not an approach that has been applied to the study of osteoarthritis associated cells and tissues, but has found utility in other complex conditions.^66^

### A Systems Biology Case Study: Mechanotransduction in Osteoarthritis

Mechanotransduction is the transfer of biomechanical forces into intracellular chemical or electrical signals and many diseases are associated with dysregulation of this activity.^67^, ^68^ OA may also be considered a disorder of mechanotransduction given that forces on the joint are integral to the health of the cartilage^69^ and evidence that OA and aged chondrocytes have altered mechanical properties.^70^, ^71^, ^72^ Biomechanical signals are also multiscale responding to age and disease, Figure 3, with effects at a tissue level (differential loading across joint, load sharing across particular tissues), within a tissue (differential compression on zonal regions of cartilage) and cell‐associated (mechanotransduction through the pericellular matrix of the chondron).^61^ Critically, there is not a single mechanical signal that transduces into an electrical or chemical signal intracellularly and different forces require a level of integration (compression, osmolarity, fluid shear, hydrostatic pressures); the contribution of each still needs to be defined.^73^ Given that mechanical signals have to be transduced through the extra‐ and peri‐cellular matrix to allow chondrocytes to respond to their physical environment mechanotransduction mechanisms are potential therapeutic targets.

Work by Guilak et al. has considered numerous modelling strategies, including FEA, to deduce mechanical responses in chondrocytes and associated peri‐cellular matrix, which helped define the complex mechanical environment consisting of changes in tension, fluid pressure/volume, shear, etc.^74^, ^75^ Using a mechanistic approach a Ca^2+^ responsive osmomechano‐TRP channel TRPV4 was found to be critical to transduction of mechanical and osmotic signals^76^ with enhanced anabolic gene expression and increased matrix production demonstrated using a chemical agonist.^73^ Further work, using a cartilage‐specific, inducible knock‐out of *Trpv4* revealed a reduction in age‐associated OA at 12 months, but not in a DMM model.^77^ This is in contrast to the severe OA phenotype observed in ageing mice with a global *Trpv4* knockout. Defining differential mechanotransduction pathways for age‐ and trauma‐associated OA could establish therapeutic targets. Modifications to a known small molecule TRPV4‐antagonist has shown analgesic and anti‐inflammatory properties that could have potential in a number of conditions including osteoarthritis.^78^ This case‐study demonstrates that a systems orientated approach (running in this case from ‘top down’) can reveal regulatory targets by modelling the integration of mechanical signals to establishing common mechanotransduction mechanisms and unravelling age‐associated contribution to biomechanical failures. By integrating clinical level mechanical and kinematic data with an understanding of cell‐level, molecular responses preventative and early therapeutic approaches may ultimately be employed in patient‐specific programmes.

### Physiology‐Based Models

As discussed earlier there are limits to the application of in vitro models of OA tissue derangement. Physiology‐based models allow perturbations to be integrated into a physiological environment so it is relevant to the scope of this review. Animal models of complex disease can facilitate a deeper understanding of the natural history of the pathology by providing controlled representations of subsets of human disease, however, there is no single standardized in vivo model and models that better represent the dynamics of human OA are required.^79^, ^80^, ^81^ Animal models build in another level of complexity, not least of which are differing temporal dynamics. Often systems biologists will use genetically simple organisms (e.g., *Caenorhabditis elegans*) to reduce the complexity of the systems under investigation. This has not been possible with OA given the particular complexity of the mammalian skeleton. However, recently some advances have been made in developing the zebrafish as a model of cartilage dysregulation.^82^, ^83^

Using developmental stages is often useful in systems orientated studies as they are conceptually simpler and easier to visualize. Depending on the model, spatiotemporal changes in expression profiles can be followed and contribute parameters to dynamic in silico simulations. Chondrogenesis, endochondral ossification, and OA pathways share regulators^84^ switching between proliferative and hypertrophic differentiation phenotypes is critical in these cases^85^, ^86^ mechanisms are employed to prevent or instigate this switch during development of articular cartilage, for example. Unlike endochondral ossification, the core regulatory network in articular cartilage development has not been resolved. It remains unclear how spatiotemporal patterns of gene expression in articular cartilage are associated with loss of function. Some studies that develop mathematical models of endochondral ossification and the balance between proliferating and hypertrophic chondrocytes have been undertaken,^87^, ^88^ but further mechanistic studies of development pertinent to an understanding of OA pathogenesis should be undertaken. Spatiotemporal expression mapping and reference atlases has been used to understand the dynamics and localization of key factors in developing tissues^89^; such an approach in joint tissues from model species would help span anatomical and molecular scales facilitating the development of cartilage expression networks and has been used in the zebrafish.^83^ There is a clear need for integration of work and tools pioneered in the field of developmental biology to be extrapolated to OA systems biology.

### Applying Systems Approaches in the Clinical Setting

We have highlighted the inherent complexity that researchers face in trying to answer the many unresolved questions in OA pathogenesis; this complexity it also demonstrable at the clinical level, not least given the multiple co‐morbidities that may be present in clinical presentations of OA. There are systems orientated approaches that may be applied to integrate mixed predictors (both qualitative and quantitative) of risk. Decision trees are one form of machine‐learning (ML) classification tools that may be applied to systems biology problems including clinical decision‐making for complex conditions.^90^ The tree structure develops from the recursive branching at binary decision points that splits a clinical data set into two mutually exclusive subsets. They are useful because they are intuitive (classification proceeds through a series of hierarchical logic questions) and are flexible in their application, being able to handle both real‐value and categorical features (e.g., biomarker levels in blood and radiographic scores) and multiple classes^91^ compared to some other forms of ML. Some examples of simple decision tree approaches have been published for clinical decision making in OA relating to imaging^92^ and arthroplasty,^93^, ^94^ but there is no evidence in the literature of more complex clinical decision trees for the classification of early osteoarthritis risk using predictors from multiple sources (e.g., imaging and biomarkers, SNPs). Further application of machine‐learning approach, such as decision trees and random forests (ensembles of decision trees) are required to deal with the multi‐scale predictors of OA risk that will emerge with systems‐orientated studies to aid clinical decision making.

### Applying Systems Approaches in the Drug Development Pipeline

Standard treatments in OA have broadly consisted of physical interventions and behavioral modifications (e.g., weight loss), pharmacological, and surgical interventions. The limitations of traditional pharmacological approaches to the symptomatic treatment of OA arise from their equivocal efficacy and/or unacceptable side‐effects. A number of next‐generation therapeutics are in clinical trial, though few have been developed to a point where regulatory approval has been granted.^22^ Exciting new approaches, such as the use of poly‐micelle protected *Runx1* mRNA,^95^ demonstrates that, in principle, articular cartilage is amenable to RNA‐based therapeutics. Given that small molecules with *Runx1*‐mediated chondroprotective properties, including *kartogenin* 96 and TD‐198946,^97^, ^98^ have been defined using high‐throughput candidate molecule screens and not systems biology approaches, can systems orientated approaches solve the problem of defining novel therapeutic targets? Systems orientated approaches augment, but do not replace reductionist strategies. They should, however, make reality the objectives of personalized medicine by understanding that network derangements, which are unlikely to be the same between individuals, are the core of complex disease pathogenesis. With respect to the indications for therapeutic use the lack of sensitive staging and phenotypic descriptors (OA phenome) means OA clinical trials will have a “one‐size‐fits‐all” approach; in demonstrating efficacy this may become problematic, requiring large and expensive trials. Systems approaches can facilitate the integration of clinical and ‘omics data, stratify clinical sub‐populations, and facilitate translation between animal and human through an understanding of shared network structure.^99^ In isolation the relative contributions of biology, structure, and mechanics may not result in OA, but rather an understanding of the interplay, and common regulatory mechanisms, between these components of joint health is required.^21^ It is likely that we need to consider therapeutic options that target multiple tissues to tackle OA, consequently, appropriate mechanistic modelling approaches to compare between cell types is required to establish therapeutic targets within signalling pathways that are relevant to both tissues.^25^ This is exemplified by the emerging discipline of systems pharmacology. Here, traditional quantitative pharmacological approaches (pharmacodynamic/kinetic models) are combined with computational modelling of the regulatory networks of the cell.^100^ This will become particularly relevant with the maturation of RNAi and CRISPR technology as therapeutic options. We have already mentioned that in complex diseases it is unlikely that a single regulatory target will suffice as a therapeutic option. As an example, a standard pharmacodynamic approach may be based on a single biomarker of interest, while an understanding of the multiple interactions of the drug with other components of a network, applicable in systems pharmacology, will help determine its efficacy.

### Systems Orientated Objectives for OA Diagnostics

High‐throughput screening has become possible with ‘omics technologies to define prognostic markers for OA (reviewed here^101^). Without a clearer understanding of the biological mechanisms involved in the aetiopathogenesis of OA the search for reliable predictors or markers of phenotypic groups would be especially challenging.^102^ Joint space narrowing is still the FDA‐approved standard for clinical efficacy and many of the other outcomes are inferred. MRI provides moderate sensitivity and there are few biochemical tests that are prognostic or diagnostic.^103^, ^104^ Currently, efforts to validate and qualify new biomarkers are focussed on further imaging and biochemical tests (Osteoarthritis Biomarker Consortium). It is notable that integrative and predictive modelling of multiscale data are not an objective for this programme. Within other drug development pipelines, for example oncology, the co‐development of companion diagnostic tests is now either common or strongly recommended.^105^ The lack of validated and specific biomarkers will retard advances in OA therapeutic development, as well as increase the cost of the associated clinical trials^106^; the potential benefit of OA therapeutics will only come from early identification of susceptible individuals and their appropriate stratification. This concurs with the work of Chu et al. who maintained that the key to prevention and treatment is the capacity to define *pre*‐osteoarthritis.^21^, ^107^ Systems approaches will encourage this type of approach to develop predictive models with diagnostic and prognostic capacity. An understanding of the interaction networks can be useful in defining similarities in phenotypes, classifying phenotypes, response to treatment, in addition to revealing potential targetable components of the cellular system.^18^ For example, in work from our group^38^ the Rho GTPase dissociation inhibitor *Arhgdib* was found to discriminate between healthy and diseased cartilage derived from the RAAK dataset.^108^ Other machine learning tools have been used for discriminatory analysis of a combination of biochemical markers, including citrullinated protein expression, between individuals with musculoskeletal disorders including early OA.^103^ As systems approaches bed down in OA research a key objective is to undertake discrimination analysis to establish genetic sub‐populations. For the part of the clinicians this requires accurate recording of phenotypic information, which is often lacking from public data repositories.

### Verification and Validation in Systems Biology

Systems biology requires considerable resources and high returns are expected. Critical appraisal of the capacity of systems biology to meet its aims in the context of OA research is required. In systems approaches where many thousands of predictions are left unverified^109^ attention to robust validation strategies is essential while reproducibility remains an unresolved issue in particular within the field of high‐throughput ‘omics. Rationale methods to verify competing models must be in place,^110^ to quantify the uncertainty in the models, ensure evidence for their application, and assess the credibility of the predictive capacity of such models. Some calls for model standardization in systems biology have been made,^111^ however, transparent publication and model sharing, release and reuse of data and code, standardized peer‐review processes and open‐source resources will be integral to progress of OA systems orientated studies. Early efforts should be made in the OA research community.

## CONCLUSIONS AND DIRECTIONS FOR FUTURE RESEARCH

In the course of the review we describe a number of approaches used by colleagues to gain a systems understanding of basic biology and OA development, but the functional output and clinical impact—changing research and clinical practice, reliable diagnostics, and disease‐modifying therapeutics—arising from these studies is not apparent at this time. The promise of systems approaches has been heralded for the last two decades as a source of new therapeutics and robust diagnostics.^112^ It is clear that this has not been the case for OA. The future success of systems orientated research in OA will rely on a number of points raised in this review. Firstly, concerted, community‐based (clinicians and researchers) approaches are required, with the use of standardized models and multi‐disciplinary teams, advances should be possible. The comprehensive collection of data, integration, discriminatory analysis, and predictive models should be a primary objective. What is becoming clear is that we do not require more bioinformatics or “dead”/static descriptions rather dynamic (“living”) mechanistic models and robust validation frameworks for models and we offer examples of approaches that, having shown utility in other disciplines, may have application in OA research (Fig. 4). We stress that modelling itself is not an end‐point for osteoarthritis research, rather it can facilitate the design of more direct and relevant experimental approaches. More subtle descriptors and development of the OA phenome, in addition to a refocusing of research strategies toward *pre‐*osteoarthritis, is critical. Clinical measurements need to be coupled to predictive models of cellular response to help direct rational intervention programmes for patients at high risk. The advent of mobile health and wearable technologies, and an understanding of social network trends on health, will facilitate collection of clinical and mechanical meta‐data to incorporate into patient‐specific models. Systems pharmacology approaches recognize that single therapeutic interventions for complex diseases are unlikely to be efficacious and insufficiently tailored to patients. RNA therapeutics will emerge as an important tool in network medicine and have the potential to promote personalized interventions in osteoarthritis. Ultimately, there is still much that is unclear about the mechanisms regulating the homeostatic system that still requires resolution before relevant multiscale models may be employed.

## AUTHOR'S CONTRIBUTIONS

AJM, MJP, CP, and PDC all revised and contributed to the article. All authors approved the final submitted manuscript.
